# Perspective on Leisure Sports Participation Among Older Adults in a Super-Aging Society: Focusing on Health Concern, Athletic Passion, Leisure Satisfaction, and Intent to Continue Participating in Leisure Sports

**DOI:** 10.3390/healthcare13010041

**Published:** 2024-12-29

**Authors:** Mun-Gyu Jun, Chulhwan Choi

**Affiliations:** 1 Department of Coaching, College of Physical Education, Kyung Hee University, Seocheon-dong 1, Giheung-gu, Yongin-si 17104, Republic of Korea; mkrollcage@khu.ac.kr; 2 Department of Physical Education, Gachon University, 1342, Seongnam-daero, Sujeong-gu, Seongnam-si 13120, Republic of Korea

**Keywords:** aging, health concern, athletic passion, leisure satisfaction, intent to continue participating in leisure sports

## Abstract

**Background/Objectives:** Today, society has a growing interest in healthy aging. This study analyzed health concerns, leisure satisfaction, athletic passion, and intention to continue participating in leisure sports of various age groups as well as the elderly, and we empirically analyzed the differences. **Methods**: This study conducted a survey of 306 adult men and women who regularly participated in leisure sports. Frequency, correlation, and difference analyses were performed using SPSS version 26.0. **Results**: The main findings indicated that older age groups showed higher levels of health concern, obsessive passion, and intention to participate in leisure sports than younger age groups, whereas younger age groups had higher levels of harmonious passion than older age groups. **Conclusions:** This study provides results through a comparative analysis that can indirectly enhance the quality of life by presenting practical alternatives to increase older adults’ participation in leisure sports activities amid the global aging trend.

## 1. Introduction

The Republic of Korea is aging faster than any other OECD member country and is expected to become a super-aging society soon. According to the aging standards established by the United Nations, a society is classified as aging when the proportion of the population aged 65 years or older reaches 7% or more of the total population. Moreover, a society is classified as an aged society when the proportion is 14% or more, whereas it is designated as a super-aging society when the proportion is 20% or more. As of 2023, the Republic of Korea is an aged society, with approximately 18.4% of its population aged 65 years or older [1]. By 2070, the global elderly population is expected to account for about 46.4% of the total population, and the Republic of Korea is also rapidly entering a super-aged society [1]. At the personal level, aging causes physical and mental health problems and leads to changes such as a reduction in social roles and income loss [2]. At the social level, it substantially burdens national economies through increased social welfare costs, such as old-age support and medical expenses [2]. Therefore, appropriate measures must be taken to prepare for this aging phenomenon.

Given that humans are finite creatures, preventing aging is impossible. Instead, it is vital to develop alternative solutions that minimize the adverse effects of aging, thereby increasing the vitality and quality of life of people who are already in an advanced age group [3]. One representative alternative is to promote physical activity and maintain a healthy mental state by having older age groups participate in sports as part of leisure activities [4]. Leisure sports activities refer to sporting activities in which individuals voluntarily participate during their daily lives or leisure, and these physical activities offer various benefits to participants, including maintaining and improving health, providing a sense of accomplishment and satisfaction, boosting confidence, and building interpersonal relationships [5,6]. Given that the benefits of leisure sports can address the physical, mental, and social challenges older adults face in an aging society, leisure sports can be utilized as a life-enhancing activity for this population.

However, participants inevitably have varying levels of satisfaction, willingness, and enthusiasm when engaging in physical activities, including leisure sports. This study focuses on four key variables—health concern, leisure satisfaction, athletic passion, and intent to continue participating in leisure sports—derived from the participation of older adults in leisure sports.

First, health concern refers to the degree of interest in one’s own health [7] and is a key motivational factor for participation in leisure activities and continued leisure participation [8]. In terms of health concern, the relationship between increased participation in leisure life and leisure activities can be observed, as both contribute to promoting healthy aging. Health concern is defined as an effort to maintain and promote health [9]. Therefore, it is a central issue for older adults, who often choose leisure activities that involve various physical activities to support healthy aging [10,11].

Second, while the primary health concern focuses on healthy aging through the physical health benefits older adults gain from participating in leisure sports activities, psychological and social wellbeing is achieved through leisure satisfaction. This concept refers to the subjective satisfaction or positive perception experienced after participating in a leisure activity [12] and is considered a key factor that influences future leisure activity choices [13]. Social and psychological satisfaction is obtained from various leisure activities, including leisure sports, and it is a major contributing factor to the choice of leisure activities [14]. Ultimately, it is a major factor that directly improves quality of life [14].

Athletic passion and the intent to continue participating in leisure sports are also relevant factors. The former refers to how actively someone participates in leisure sports activities, whereas the latter indicates how long one will continue participating in leisure sports activities. First, athletic passion refers to a strong inclination to devote substantial time and energy to a sport that one loves and values, and it is defined as an attitude of strong drive toward a pursued goal with enthusiasm [15]. Athletic passion is classified into harmonious and obsessive depending on whether it is voluntary or compulsive (i.e., due to internal pressure). Harmonious passion is characterized by feelings of vitality when engaging in voluntarily chosen activities, allowing individuals to participate with psychological stability. In contrast, obsessive passion arises from internal pressure, compelling someone to engage in a specific activity, and is perceived as a situation where the individual has no choice but to participate [16]. Second, intent to continue participating in leisure sports refers to an individual’s determination and mindset to consistently engage in their chosen form; continuous participation in leisure sports serve as a source of strength, contributing to health maintenance and stress relief, as well as offering enjoyment, leisure satisfaction, and self-realization [17]. Older adults may have difficulty participating in certain leisure activities, regardless of their voluntary intentions, because of their relatively degenerated physical abilities [18,19]. If an activity is judged not to meet their needs, this age group may exhibit a lower participation rate and enthusiasm level than other age groups [18,19]. Therefore, older adults exhibit varying levels of health concern, leisure satisfaction, athletic passion, and intent to continue participating in leisure sports. Therefore, it could be essential to propose alternatives for enjoying leisure sports more effectively by studying these topics.

Among the many personal characteristics that determine the key variables related to leisure sports participation, this study aimed to examine differences by age group. Older adults change their physical activity preferences slowly as they progress through physical and mental aging [20]. Moreover, as people move into the early, middle, and late aging stages, their lifestyles change, and their leisure pursuits tend to change accordingly [21]. However, no studies have evaluated whether the various variables associated with participation in leisure sports activities differ by age group among older adults. In light of this, examining the differences in key variables associated with leisure sports participation among different age groups is significant.

In summary, this study aimed to empirically analyze differences in health concern, leisure satisfaction, athletic passion, and the intent to continue participating in leisure sports across all age groups participating in leisure sports in the Republic of Korea. The results will serve as a foundation for developing strategies that can indirectly enhance the quality of life for older adults by presenting practical alternatives to increase their participation in leisure sports activities amid the global aging trend. The following four hypotheses were tested via comparative analysis in this study:

**Hypothesis** **H1.** *Health concern differs according to the age of leisure sports participants.*

**Hypothesis** **H2.** *Athletic passion differs based on the age of leisure sports participants.*

**Hypothesis** **H3.** *Leisure satisfaction differs according to the age of leisure sports participants.*

**Hypothesis** **H4.** *Intent to continue participating in leisure sports differs based on the age of leisure sports participants.*

## 2. Materials and Methods

### 2.1. Data Collection Procedure

Participants were South Korean adults aged 20 and older who regularly participated in leisure sports. After the approval of the Institutional Review Board (No. 1044396-202405-HR-077-01), a quantitative research survey method was used to collect data over a two-month period, from June to July 2024. The convenience sampling method, a type of non-probability sampling, was used to gather data in accordance with participant eligibility criteria. An online survey platform provided by Google was used. This study granted the qualification to participate in the survey to Koreans who responded that they regularly participated in leisure sports more than once a week. Participation in the survey was limited by minors under the age of 20. Based on the convenience sampling, online surveys were conducted at three sports facilities located in Seoul and two local community centers located in the metropolitan area. Only those who expressed their intention to participate were sent a link to the survey through a smartphone. Respondents read a brief description of the purpose of this study and voluntarily completed the self-administered questionnaire including online informed consent. Three hundred and six copies of the questionnaires were collected.

### 2.2. Research Tools

This study measured participants’ demographic characteristics, health concern, athletic passion, leisure satisfaction, and intent to continue participating in leisure sports. The measurement tools used were as follows.

First, the demographic characteristics considered were gender, age group, mean monthly income, years of participation in leisure sports, frequency of participation in leisure sports per week, and type of leisure sports. These variables were measured using six nominal scales.

Second, health concern was measured using a five-item single-factor scale developed by Park et al. [22]. The tool consists of items that measure participants’ interest in health-related knowledge, health-related behaviors, and health-related attitudes. Cronbach’s alpha at the time of development was 0.74. This study used the tool on a 5-point Likert scale and calculated mean scores; a higher mean score indicated higher health concern.

Third, the passion scale developed by Vallerand et al. [23] and modified and validated by Marsh et al. [24] was used to measure athletic passion. The original version consists of six items for harmonious passion and six items for obsessive passion. This study shortened and reconstructed them into three items each and then modified them into items related to participants’ participation in leisure sports. Marsh et al. [24] reported that Cronbach’s alpha was 0.83 for harmonious passion and 0.86 for obsessive passion. This study measured the items using a 5-point Likert scale; higher mean scores indicated higher levels of harmonious/obsessive passion.

Fourth, leisure satisfaction was measured using the leisure satisfaction scale developed by Beard and Ragheb [12] and validated by Kang [25]. The original tool consisted of a single factor scale with four items to capture differences between age groups more succinctly by measuring satisfaction with participation in leisure sports as a single dimension. Kang [25] reported that Cronbach’s alpha ranged from 0.80 to 0.92 for the original tool. This study used the tool based on a 5-point Likert scale and interpreted a higher mean score as indicating a higher level of leisure satisfaction.

Finally, the study utilized a tool developed by Jeong [26] to measure Korean adults’ motivation to participate in leisure sports, which was validated by Lee [27] to measure the intent to continue participating in leisure sports. The tool measures participants’ intent to continue participating in leisure sports using six items across three sub-categories (intention to participate, importance intention, and abulia). This study reorganized it into two sub-categories (intention to participate and abulia) because the importance intention sub-category is identical to intention to participate. In this study, the term abulia was defined as a state of psychological status lacking motivation; drive; or willingness to act, speak, or think [27]. The previous study [27] reported a Cronbach’s alpha of 0.68 for intention to participate, 0.52 for importance intention, and 0.91 for abulia. This study used the tool based on a 5-point Likert scale. A higher mean score for each sub-category was interpreted as a higher level of intent to participate or abulia in leisure sports.

### 2.3. Statistical Analysis Method

We analyzed 306 valid responses collected in this study using SPSS version 26.0. First, a frequency analysis was conducted to identify the participants’ basic characteristics. Second, exploratory factor analysis was conducted, and Cronbach’s alpha was calculated to test the validity and reliability of the measurement tools. Third, a descriptive statistical analysis was conducted to calculate the mean and standard deviation of the main concepts of this study, which were derived from the previous validation results. A Pearson’s correlation analysis was performed to examine the relationships between the main variables. Lastly, a one-way ANOVA and Scheffe’s post hoc test were conducted to test the differences in health concern, athletic passion, leisure satisfaction, and intent to continue participating in leisure sports by age group. The significance level for all statistical analyses was set at *p* < 0.05.

## 3. Results

### 3.1. Participants’ Basic Characteristics

Table 1 shows the distribution of the participants’ basic characteristics. The study included 159 men (52.0%) and 147 women (48.0%). The largest age group was over 66 years (111 participants, 36.3%), followed by 46–65 years (100 participants, 32.7%), and 20–45 years (95 participants, 31.0%). In terms of mean monthly income, most participants (72, 23.5%) earned between KRW 1 million and KRW 3 million. Moreover, 82 participants (26.8%) had participated in leisure sports for more than 20 years, the highest number in this category. Among the participants, 112 (36.6%) engaged in leisure sports 1–2 times per week, which was the most common participation pattern, and 244 participants (79.7%) engaged in individual sports, while 62 (20.3%) engaged in team sports.

### 3.2. Validity and Reliability of Research Tools

The main observed variables were health concern, athletic passion (harmonious and obsessive), leisure satisfaction, and intent to continue participating in leisure sports (importance intention, intention to participate, and abulia). Exploratory factor analysis was conducted to verify the validity of each observed variable, and Cronbach’s α, an internal reliability coefficient, was calculated to examine the reliability of the research tools. The varimax rotation method was used for factor analysis and principal component extraction. Each item was named “Health Concern 1”, “Health Concern 2”, and so on according to the order of the items, and four factor analyses were conducted for each variable.

#### 3.2.1. Health Concern

Table 2 presents the results of the factor analysis conducted on the tool used to measure health concern. The original tool consists of five items related to health concern. The initial factor analysis yielded an intact single factor without excluding any items. The factor was named “Health Concern”, and the validity of the factor classification was secured because the factor loadings of the five items were above 0.6, and the eigenvalue of the single factor was 3.373, which was much higher than 1. The Cronbach’s *α* of Health Concern, the single factor, was 0.875, which was higher than 0.6, proving that it was a reliable tool.

#### 3.2.2. Athletic Passion (Obsessive and Harmonious)

Table 3 shows the factor analysis results for the tool used to measure athletic passion. The original tool classifies athletic passion into obsessive and harmonious, each consisting of three items. The initial factor analysis also identified two factors without dropping any items. Therefore, Factor 1, which comprises items about harmonious passion, was named “Harmonious Passion”, while Factor 2, which comprises questions about obsessive passion, was named “Obsessive Passion”. 

The validity of the factor classification was confirmed because the factor loadings of all items comprising each factor were above 0.6 and the eigenvalues of the two factors were above 1. The Cronbach’s *α* for each factor was then calculated. Cronbach’s *α* of Harmonious Passion was 0.946, and that of Obsessive Passion was 0.895, which were higher than 0.6, proving that it is a reliable tool.

#### 3.2.3. Leisure Satisfaction

Table 4 presents the results of the factor analysis of the tool used to measure leisure satisfaction. The original tool consists of four items related to leisure satisfaction, and the initial factor analysis resulted in an intact single factor without any items being dropped. Therefore, the factor was named “Leisure Satisfaction”. The validity of the factor classification was secured because the factor loadings of all four items were above 0.6 and the eigenvalue of the single factor was 3.369, which was much higher than 1. Cronbach’s *α* for Leisure Satisfaction, the single factor, was 0.936, which exceeds 0.6, proving that it is a reliable tool.

#### 3.2.4. Intention to Continue Participating (Intention to Participate and Abulia)

Table 5 shows the factor analysis results of the tool used to measure intent to continue participating in leisure sports. This study reclassified the participants’ intent to continue participating in leisure sports into intention to participate and abulia, and factor analysis resulted in two factors, abulia and intention to participate, without any items being dropped. Therefore, Factor 1, items on abulia, was named “Abulia”, and Factor 2, items on intention to participate, was named “Intention to Participate”.

The validity of the factor classification was confirmed because the factor loadings of all items comprising each factor were above 0.6 and the eigenvalues of the two factors were above 1. The Cronbach’s *α* for each factor was then calculated. Cronbach’s *α* for Abulia was 0.928, and that for Intention to Participate was 0.923, which were higher than 0.6, proving that it is a reliable tool.

### 3.3. Descriptive Statistics and Correlation Analysis for Multicollinearity

Table 6 shows the descriptive statistics and results of the correlation analysis of the main variables, which were selected based on the results of the previous validity and reliability tests.

First, the descriptive statistics analysis showed that the mean scores for the main variables ranged from 2.16 (Intention to Continue Participating: Abulia) to 3.91 (Leisure Satisfaction). Moreover, their absolute skewness and kurtosis values were less than 3 and 10, respectively. Therefore, these variables satisfied the normality assumption that the responses of the participants would be similar to those of the population. Therefore, this study has established the feasibility of using parametric statistical techniques.

Second, correlation analysis between variables was performed to confirm multicollinearity using the Pearson correlation coefficient. Multicollinearity was statistically satisfied in that all the result values were lower than 0.700.

### 3.4. Differences in the Level of Health Concern, Athletic Passion, Leisure Satisfaction, and Willingness to Continue Participating in Leisure Sports by Age Group

Finally, a one-way ANOVA and post hoc test were conducted to test the differences in the mean scores of the key variables by age group; the results are shown in Table 7. The results revealed that there was statistically significant age difference on Health Concern (F = 6.582, *p* < 0.01), Harmonious Passion (F = 33.353, *p* < 0.001), Obsessive Passion (F = 8.044, *p* < 0.001), and the Intention to Participate (F = 7.769, *p* < 0.01). However, Leisure Satisfaction and Abulia did not differ significantly by age group. Post hoc tests showed that older age groups had higher levels of Health Concern, Obsessive Passion, and Intention to Participate than younger age groups. In contrast, younger age groups showed higher levels of Harmonious Passion, a subcomponent of Athletic Passion, than older age groups.

## 4. Discussion

Aging and low birth rates have been recognized as significant social concerns, contributing to a range of social problems [28]. With advancements in the medical industries and improvements of living environment, life expectancy is increasing, while the fertility rate is plunging due to shifts in social structures and changing perceptions [29]. Consequently, society must now adapt to the growing challenge of population aging. There may be various ways to address these changes; however, this study aimed to compare and analyze health concern, athletic passion, leisure satisfaction, and intent to continue participating across different age groups, particularly in the context of the rapidly increasing older adult population in the Republic of Korea. The goal is to better understand the characteristics of older adults and develop objective, appropriate suggestions for promoting healthier lifestyles among older adults, in comparison with other age groups. Older adults’ participation in leisure sports is currently recognized as an irreplaceability rather than an option, and the concept of leisure itself is regarded as an area of welfare [30]. This means that the creation of an environment in which all older adults can participate in leisure sports should be considered at the governmental level [31]. This study analyzes the perceptions of older adults’ on maintaining a healthy life through leisure sports in comparison to other age groups. It is essential to identify the characteristics of older adults and to offer appropriate suggestions tailored to them. The discussion of the results derived from this study is as follows.

First, the results showed that health concern, adopted as a major dependent variable in this study, was statistically higher in the oldest age group than in the other two groups. This can be attributed to the super-aging society that has emerged in recent years. Advances in medical technology have led to increased life expectancy [29], which, in turn, has heightened interest in and attention to health. In addition, in a more diversified media environment, people now access significantly more health information than in the past, contributing to increased interest in the health of older adults [32]. All humans experience aging. Relatively older people who are more aware of this process are likely to take actions to improve their health, informed by the vast amount of health information available. In this study’s questionnaire, health concern broadly included responsibility for personal health, including food, physical activity, and weight management. A recent study by Kim [33] showed that individuals in higher age groups were more willing to spend on health promotion through physical activities than those in other age groups. This willingness to spend was not necessarily due to higher income but rather increased interest in health. As such, the interest among older adults in delaying aging and leading a healthy life is stronger than ever. Therefore, various leisure sports and welfare programs tailored to older adults should be implemented.

Next, the comparative analysis of athletic passion (i.e., harmonious passion and obsessive passion), which was adopted as a key dependent variable in this study, revealed statistically significant differences between the three age groups. Specifically, regarding harmonious passion, the two younger age groups showed higher scores. This result suggests that the younger generation, who may not yet be concerned with concepts such as life expectancy and aging, tend to pursue physical activities for diverse experiences and values rather than for physical health alone. Recently, the younger generation tends to enjoy various sports rather than consistently participating in one leisure sports event [34]. In addition, some argue that the younger generation prefers to form and maintain new social relationships by participating in leisure sports [35]. In contrast, the obsessive passion factor focuses on health promotion itself through physical activities. Consequently, obsessive passion was higher in the relatively older age group. This suggests that older adults prioritize health improvement over seeking new and diverse experiences in their leisure activities. Based on the concept of obsession, participation in leisure sports is accompanied by a high level of enthusiasm [36]. In addition, health maintenance among older adults may be relatively highly dependent on participation in leisure sports compared to that of the younger generation. This shows that older adults view physical activity through leisure sports as essential rather than optional [37]. Therefore, current policies that include participation in leisure sports as part of welfare programs are moving in the right direction.

Additionally, a comparative analysis of age regarding the factors influencing the intent to continue participating in leisure sports (specifically, intent to participate and abulia) revealed statistically significant results for the intention to participate. This intention reflects a psychological commitment to engage in leisure sports consistently over time. Furthermore, the concept of intention implies an active psychological determination to pursue physical activities, despite internal and external constraints [38]. In this study, the relatively older age group exhibited higher mean scores, thus confirming their willingness to participate in leisure sports. This suggests that their interest in health and compulsive athletic passion, as analyzed earlier, also translates into a commitment to continued participation. It was confirmed that older adults’ intent to engage in leisure sports was driven by long-term health benefits, not by temporary interest or enthusiasm. Generally, the benefits of physical activity extend beyond physical health to include mental and social improvements [39]. Participating in physical activities may serve as a fundamental solution for older adults experiencing psychological and social role changes due to physical aging [40]. This study underscores the increasing need to examine leisure sports participation from various age-specific perspectives. Ultimately, to encourage sustained participation in leisure sports, it is necessary to develop tailored physical activities based on age. Such age-specific programs may provide optimal opportunities for older individuals who are willing to maintain an active lifestyle.

Among the six variables in this study, four variables (e.g., health concern, harmonious passion, obsessive passion, and intention to participate) showed significant differences between groups and two (e.g., leisure satisfaction and abulia) did not. Among the two variables that did not show a significant difference, leisure satisfaction showed the highest mean score for all survey respondents in this study regardless of the group differences: (a) health concern (*M* = 3.80), (b) harmonious passion (*M* = 3.62), (c) obsessive passion (*M* = 3.33), (d) leisure satisfaction (*M* = 3.91), (e) intention to participate (*M* = 3.67), and (f) abulia (*M* = 2.15). In other words, regardless of age, participants provided the strongest support for leisure satisfaction. These findings are significant in the context of a social climate where interest in health has recently increased and satisfaction after participating in leisure sports was the highest. Satisfaction has been defined as an evaluation of the outcome, and subjective psychological evaluation should ultimately be judged based on the expectations established before the action [41]. In conclusion, people who already recognize the general understanding that leisure sports positively affect health reported higher levels of satisfaction. In other words, despite the already high expectations, satisfaction with participation in leisure sports remains the highest. Previous studies [42] have shown that participation in leisure sports not only guarantees physical benefits but also brings mental and social benefits. Furthermore, satisfaction is regarded as the most important factor because psychological satisfaction has the most significant influence on the factors of motivation that determine people’s behavior and will determine continuous behavior [43]. The results of this study will be an opportunity to once again establish the elderly’s perception of participation in leisure sports.

## 5. Conclusions

This study analyzed health concerns, leisure satisfaction, athletic passion, and intention to continue participating in leisure sports of various age groups as well as the elderly, and we empirically analyzed the differences. The comparative analysis provides results that can indirectly enhance the quality of life by presenting practical alternatives to increase older adults’ participation in leisure sports activities amid the global aging trend. In spite of the contributions of this study there are some limitations to be addressed in future studies.

First, it examined differences in key concepts related to leisure sports participation across various age groups. Future studies should analyze the causal relationships that influence the intention to continue participating in leisure sports at different life stages and compare the effects of specific sub-factors (e.g., leisure participation motivation, constraints, and negotiation). Future studies may have implications for leisure sports participation across all age groups, not just older adults, because they may identify the factors that most strongly influence the intention to participate in leisure sports for each age group, going beyond this study’s focus on identifying simple differences. Second, this study is likely subject to errors of generalization because it examined differences in key leisure-sports-related variables across age groups based on the survey responses of 306 participants in the Republic of Korea. Future studies should draw more objective conclusions by conducting large-scale studies covering a variety of regions, time periods, and age groups.

## Figures and Tables

**Table 1:** Participants’ basic characteristics (n = 306).

Classification		Group 1 (n = 95) (20–45 Years Old)	Group 2 (n = 100) (46–65 Years Old)	Group 3 (n = 111) (over 66 Years Old)
		Frequency (%)		
Gender	Male	39 (41.1)	59 (59.0)	61 (55.0)
	Female	56 (58.9)	41 (41.0)	50 (45.0)
Mean monthly income	Less than USD 800	42 (44.2)	5 (5.0)	5 (4.5)
	USD 800–2000	45 (47.4)	14 (14.0)	13 (11.7)
	USD 2000–3500	6 (6.3)	32 (32.0)	26 (23.4)
	USD 3500–5000	1 (1.1)	11 (11.0)	35 (31.5)
	USD 5000 or more	1 (1.1)	38 (38.0)	32(28.8)
Years of participation in leisure sports	Less than 5 years	39 (41.1)	25 (25.0)	17 (15.3)
	5–10 years	30 (31.6)	14 (14.0)	22 (19.8)
	10–15 years	25 (26.3)	10 (10.0)	7 (6.3)
	15–20 years	1 (1.1)	16 (16.0)	18 (16.2)
	20 years or more	-	35 (35.0)	47 (42.3)
Frequency of participation in leisure sports	Once a week	23 (24.2)	38 (38.0)	14 (12.6)
	1–2 times per week	29 (30.5)	39 (39.0)	44 (39.6)
	3–4 times per week	2 (2.1)	2 (2.0)	12 (10.8)
	5 times per week	41 (43.1)	21 (21.0)	41 (38.9)
Type of leisure sports	Individual sports	79 (73.2)	89 (89.0)	76 (68.5)
	Team sports	16 (16.8)	11 (11.0)	35 (31.5)
Total		306 (100)		

**Table 2:** The validity and reliability of the health concern measurement tool.

Items	Factor Loading	Cronbach’s *α*
	1	
Health Concern 2	0.880	0.875
Health Concern 1	0.874	
Health Concern 4	0.834	
Health Concern 3	0.796	
Health Concern 5	0.711	
Factor Name	Health Concern	
Eigenvalue	3.373	
Individual Proportion of Variance	67.459	
Cumulative Proportion of Variance	67.459	

KMO measure: 0.847, Bartlett’s unit matrix test: 806.458 (*p* < 0.001).

**Table 3:** The validity and reliability of the athletic passion measurement tool.

Items	Factor Loading		Cronbach’s *α*
	1	2	
Athletic Passion—(harmonious) 1	0.947	0.079	0.946
Athletic Passion—(harmonious) 2	0.947	0.153	
Athletic Passion—(harmonious) 3	0.940	0.111	
Athletic Passion—(obsessive) 2	0.090	0.926	0.895
Athletic Passion—(obsessive) 3	0.047	0.897	
Athletic Passion—(obsessive) 1	0.202	0.884	
Factor Name	Harmonious Passion	Obsessive Passion	
Eigenvalue	2.727	2.487	
Individual Proportion of Variance	45.448	41.444	
Cumulative Proportion of Variance	45.448	86.892	

KMO measure: 0.770, Bartlett’s unit matrix test: 1482.799 (*p* < 0.001).

**Table 4:** The validity and reliability of the leisure satisfaction measurement tool.

Items	Factor Loading	Cronbach’s *α*
	1	
Leisure Satisfaction 4	0.942	0.936
Leisure Satisfaction 2	0.927	
Leisure Satisfaction 3	0.902	
Leisure Satisfaction 1	0.899	
Factor Name	Leisure Satisfaction	
Eigenvalue	3.369	
Individual Proportion of Variance	84.213	
Cumulative Proportion of Variance	84.213	

KMO measure: 0.855, Bartlett’s unit matrix test: 1083.278 (*p* < 0.001).

**Table 5:** The validity and reliability of the intent to continue participating measurement tool.

Items	Factor Loading		Cronbach’s *α*
	1	2	
Intent to Continue Participating—(abulia) 2	0.948	−0.014	0.928
Intent to Continue Participating—(abulia) 3	0.937	0.042	
Intent to Continue Participating—(abulia) 1	0.918	−0.097	
Intent to Continue Participating—(participation) 1	−0.008	0.946	0.923
Intent to Continue Participating—(participation) 2	−0.045	0.928	
Intent to Continue Participating—(participation) 3	−0.015	0.918	
Factor Name	Abulia	Intention to participate	
Eigenvalue	2.621	2.610	
Individual Proportion of Variance	43.677	43.496	
Cumulative Proportion of Variance	43.677	87.173	

KMO measure: 0.738, Bartlett’s unit matrix test: 1438.677 (*p* < 0.001).

**Table 6:** Descriptive statistics and correlations between key variables.

Classification		Health Concern	Athletic Passion		Leisure Satisfaction	Intent to Continue Participating	
			Harmonious Passion	Obsessive Passion		Abulia	Intent to Participate
Health Concern		1					
Athletic Passion	Harmonious Passion	0.198 ***	1				
	Obsessive Passion	0.514 ***	0.254 ***	1			
Leisure Satisfaction		0.353 ***	0.370 ***	0.486 ***	1		
Intent to Continue Participating	Abulia	−0.192 **	−0.375 ***	−0.250 ***	−0.409 ***		
	Intent to Participate	0.447 ***	0.050	0.409 ***	0.204 ***	−0.049	1
Mean (M)		3.80	3.62	3.33	3.91	2.16	3.67
Standard Deviation (SD)		0.787	1.075	0.990	0.755	0.976	1.119
Skewness		−0.473	−0.261	−0.187	−0.186	0.924	−0.914
Kurtosis		−0.164	1.077	−0.607	−0.637	0.792	0.177

** *p* < 0.01, *** *p* < 0.001.

**Table 7:** Differences in dependent variables by age.

Classification		Dependent Variables (M ± SD)					
		Health Concern	Athletic Passion		Leisure Satisfaction	Intent to Continue Participating	
			Harmonious Passion	Obsessive Passion		Abulia	Intent to Participate
Age Groups	20–45 years (n = 95): a	3.67 ± 0.742	3.93 ± 0.811	3.20 ± 0.984	3.92 ± 0.743	2.12 ± 0.907	3.47 ± 1.174
	46–65 years (n = 100): b	3.68 ± 0.744	4.00 ± 0.682	3.14 ± 1.026	3.90 ± 0.677	2.12 ± 1.075	3.49 ± 1.246
	Over 66 years (n = 111): c	4.01 ± 0.823	3.02 ± 1.283	3.62 ± 0.897	3.91 ± 0.834	2.23 ± 0.945	4.00 ± 0.853
F(*p*)		6.582 ** (0.002)	33.353 *** (0.000)	8.044 *** (0.000)	0.030 (0.970)	0.437 (0.646)	7.769 ** (0.001)
Post Hoc Test		a, b < c	a, b > c	a, b < c	-	-	a, b < c

** *p* < 0.01, *** *p* < 0.001.

## Data Availability

The original contributions presented in the study are included in the article; further inquiries can be directed to the corresponding author.
